# Associations between high-density lipoprotein cholesterol, non-high-density lipoprotein cholesterol, and their ratio with metabolic dysfunction-associated steatotic liver disease: a retrospective cohort study

**DOI:** 10.3389/fendo.2025.1585811

**Published:** 2025-06-18

**Authors:** Hong Chen, Xinlei Miao, Manling Hu, Ziping Song, Yangxuan He, Jiayi Deng, Song Leng

**Affiliations:** ^1^ Health Management Center, The Second Affiliated Hospital of Dalian Medical University, Dalian, Liaoning, China; ^2^ Department of General Medicine, The Second Affiliated Hospital of Dalian Medical University, Dalian, Liaoning, China; ^3^ Department of Gastroenterology, The Second Affiliated Hospital of Dalian Medical University, Dalian, Liaoning, China; ^4^ School of Public Health, Dalian Medical University, Dalian, Liaoning, China

**Keywords:** non-high-density lipoprotein cholesterol to high-density lipoprotein cholesterol ratio, non-high-density lipoprotein cholesterol, high-density lipoprotein cholesterol, metabolic dysfunction-associated steatotic liver disease, retrospective study

## Abstract

**Objective:**

High-density lipoprotein cholesterol (HDL-C), non-HDL-C, and the ratio of non-HDL-C to HDL-C (NHHR) are closely correlated with multiple metabolic diseases. This study aims to dissect their associations and differences in relation to new-onset MASLD.

**Methods:**

Data were collected from research subjects at the Health Management Center of the Second Affiliated Hospital of Dalian Medical University between 2014 and 2023. Participants were stratified by quartiles of HDL-C, non-HDL-C, and NHHR. Kaplan–Meier analysis, Cox proportional hazards models, restricted cubic splines (RCS), sensitivity analyses, and receiver operating characteristic (ROC) curves were employed to evaluate associations between NHHR, non-HDL-C, HDL-C, and new-onset MASLD and compare predictive performance across lipid parameters.

**Results:**

A total of 36,897 participants (mean age 42.1 years; 56.5% female) were followed for a mean of 3.19 years, with 20.3% developing new-onset MASLD. Cox regression showed that compared to the Q1 group, the Q4 group of NHHR and non-HDL-C had a 134% (HR=2.34, 95% CI: 2.13–2.56) and 22% (HR=1.22, 95% CI: 1.13–1.31) higher risk of MASLD, respectively, while HDL-C was associated with a 45% lower risk (HR=0.55, 95% CI: 0.50–0.60). RCS analysis demonstrated nonlinear relationships for NHHR (threshold = 2.54) and HDL-C (threshold = 1.31 mmol/L), whereas non-HDL-C displayed a linear, positive association with MASLD risk. Stratified analyses revealed that elevated non-HDL-C levels conferred higher MASLD risk in men, whereas females, younger adults, and individuals with lower cardiometabolic burden (BMI <24 kg/m², nonhypertensive, and nonhyperuricemic status) showed steeper increases in MASLD risk with rising NHHR quartiles. ROC analysis indicated NHHR was superior to other lipid parameters in predicting MASLD risk.

**Conclusion:**

Decreases in HDL-C levels and increases in non-HDL-C and NHHR levels may increase the risk of MASLD. The NHHR can be used as a new index that is stronger than other lipoproteins for the prediction of MASLD.

## Introduction

Metabolic dysfunction-associated steatotic liver disease (MASLD), formerly known as nonalcoholic fatty liver disease (NAFLD) or metabolism-associated fatty liver disease (MAFLD), has become a global public health problem (1). Characterized by cardiometabolic risk factors such as obesity, type 2 diabetes, hypertension, and dyslipidemia, MASLD is a chronic disease involving multiple systems and is closely associated with insulin resistance and genetic predisposition; and is linked to higher rates of illness and death due to cardiovascular disease, chronic kidney disease, and various cancers (2, 3). However, there are no approved drugs for the specific treatment of MASLD, and with a current global prevalence reaching 38%, it is becoming the most common cause of chronic liver disease (4). Moreover, China is expected to have the fastest growing total number of MASLD cases in the future (5).

Some traditional and nontraditional lipid parameters, such as triglyceride-glucose (TyG), non-high-density lipoprotein cholesterol (non-HDL-C), low-density lipoprotein cholesterol (LDL-C), high-density lipoprotein cholesterol (HDL-C), remnant cholesterol (RC), and related composites, have good predictive value for MASLD (6–8), allowing early identification of high-risk patients and avoiding the risks associated with liver biopsy. In recent years, an increasing number of new composite atherogenic lipid indicators have been used to assess various metabolic diseases. One such indicator is the non-HDL-C/HDL-C ratio (NHHR). Low serum HDL-C levels, a component of the NHHR, have historically been linked to MASLD, yet recent studies have shown that high HDL-C levels may increase atherosclerosis and the incidence of cardiovascular disease (9, 10). Similar to HDL-C, a prospective study from the National Health and Nutrition Examination Survey reported a U-shaped correlation between non-HDL-C and both all-cause mortality and cardiovascular mortality in men (11). Thus, the relationships between NHHR components and MASLD still need further investigation. The NHHR has been proposed to be a stronger predictor than non-HDL-C of a variety of metabolic diseases, including adverse cardiovascular events, thyroid hormones, hyperuricemia, diabetes, and periodontitis (12–16). Furthermore, it is considered a crucial prognostic factor for cardiovascular disease (CVD), the leading cause of death in individuals with MASLD (17). Most existing studies linking NHHR to MASLD are cross-sectional (18–20), highlighting the need for longitudinal analyses to confirm these associations.

Therefore, given the close associations of HDL-C, non-HDL-C, and the NHHR with many metabolic diseases, this retrospective study investigated their associations with new-onset MASLD and differences in predictive ability for MASLD on the basis of a large ongoing health screening cohort, with the aim of providing a reference for the prevention of MASLD and other metabolic diseases.

## Materials and methods

### Study population

**Figure 1:**
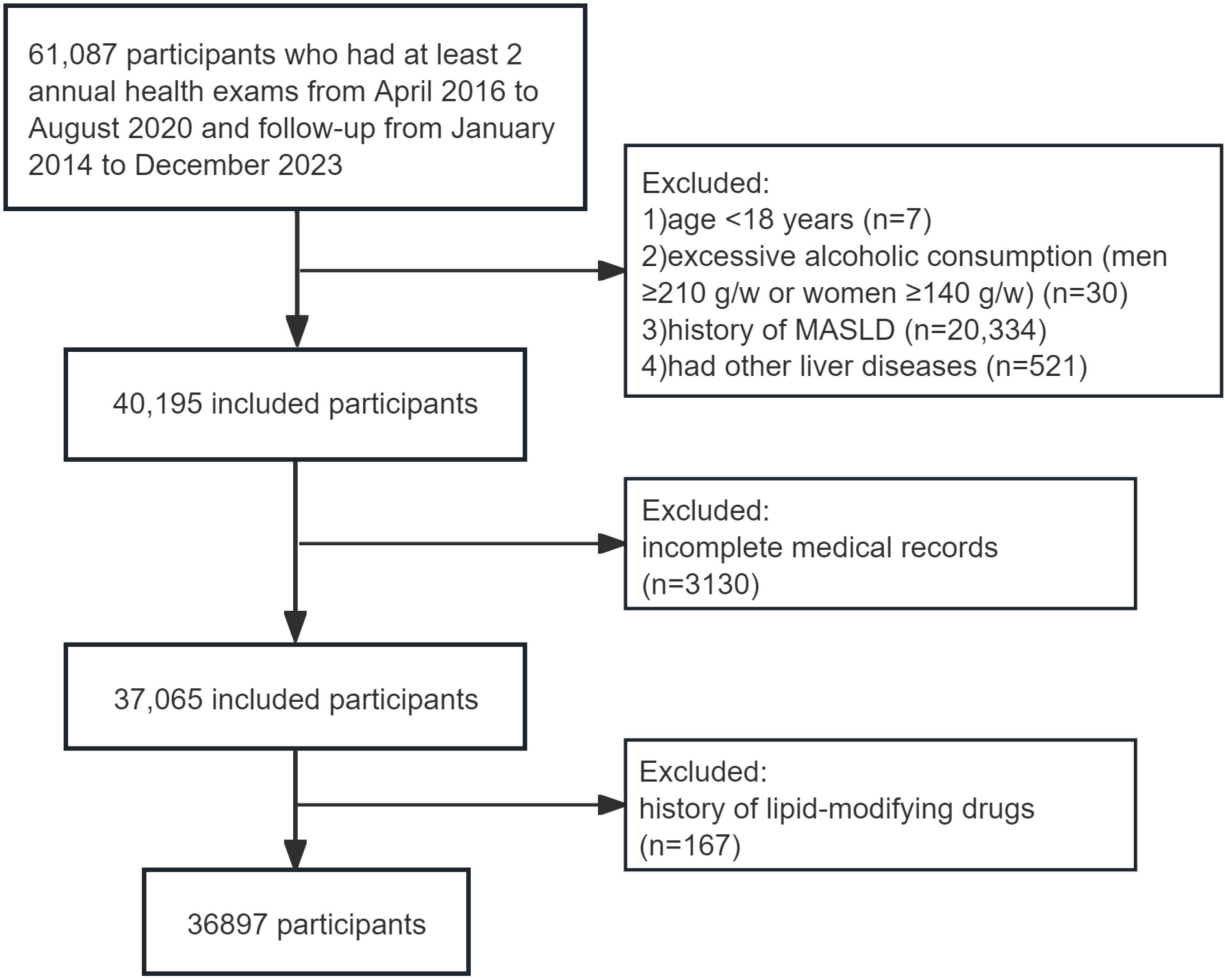
Participant flow diagram.

The population was drawn from the Dalian Health Management Cohort (DHMC) (ChiCTR2300073363) at the Second Hospital of Dalian Medical University. This cohort collected key longitudinal physical examination results of the population, evaluated their health status, and provided further health management. A total of 61,087 participants who had at least 2 annual health exams between January 2014 and December 2023 were included in this dynamic cohort study, and the following exclusion criteria were used: (a) age < 18 years (n=7); (b) excessive alcoholic consumption, defined as consuming ≥210 g per week for men or ≥140 g for women (n=30); (c) diagnosed with MASLD (n=20,334), viral hepatitis, autoimmune hepatitis, cirrhosis or cancer at baseline (n=521); (d) missing total cholesterol (TC) or HDL-C data (n=1232), missing the diagnostic indicators of MASLD (n=1,898); and (e) treatment with lipid-modifying drugs for at least 6 months before the baseline survey (n=167). Finally, 36897 subjects who met the criteria were included in this study (**Figure 1**). Baseline data were defined as the earliest physical examination data from each participant. People were followed up to the time when MASLD was diagnosed or the time of the last visit, whichever was earlier. The study received approval from the Ethics Committee of the Second Hospital of Dalian Medical University (grant number: 2,022,064), which waived the requirement for patient informed consent. The study was performed in accordance with the Declaration of Helsinki.

### Clinical and laboratory variables

Demographic information, including gender, age, and medical history, was collected using standardized self-management questionnaires. The participants wore light clothing, and their height and weight were measured while they were barefoot. Waist circumference was measured by trained nurses at the midpoint between the lower rib margin and the iliac crest. BMI was computed as weight (kg) divided by height (m^2^). The Omron electronic blood pressure monitor (HBP-9020, Japan) was employed to measure blood pressure after a five-minute rest. After fasting for at least 8 hours, hematological samples were collected in the morning, and a Roche Cobas C 501 Chemistry Analyzer was used to measure gamma-glutamyl transpeptidase (GGT), triglyceride (TG), fasting plasma glucose (FPG), HDL-C, LDL-C, alanine aminotransferase (ALT), TC, aspartate aminotransferase (AST), albumin (ALB), uric acid (UA), creatinine (Cr), total bilirubin (TBil), direct bilirubin (DBil), and blood urea nitrogen (BUN) levels. Platelet count (PLT), hemoglobin (Hb), and white blood cell count (WBC) were analyzed using a Mindray BC-6900CRP automated hematology analyzer.

Non-HDL-C = TC-HDL-C. TyG index = ln (fasting triglycerides (mmol/L) ×88.6×FPG (mmol/L) ×18/2). NHHR = non-HDL-C/HDL-C.

Hypertension was defined as a systolic blood pressure (SBP)≥140 mmHg and/or diastolic blood pressure (DBP)≥90 mmHg, the use of antihypertensive drugs, or self-reported diagnosis (21). Diabetes mellitus was diagnosed based on insulin or hypoglycemic medication use, a diabetes history, or FPG ≥7 mmol/L (22). Hyperuricemia was defined as UA >417 μmol/L (7.0 mg/dL) in men and >357 μmol/L (6.0 mg/dL) in women (23).

### Diagnostic criteria for MASLD

MASLD was diagnosed by experienced ultrasonographers using a Siemens ACUSON Sequoia Silver ultrasound system. The diagnostic criteria aligned with the modified Delphi approach of the MASLD consensus (1), requiring ≥5% steatosis, excluding other causes or excessive alcohol intake (≥20 g/d for women, ≥30 g/d for men), with at least one cardiometabolic risk factor: (1) BMI ≥23 kg/m^2^ or waist circumference ≥90/80 cm (men/women); (2) FPG ≥5.6 mmol/L, hemoglobin A1c ≥5.7%, or T2DM/treatment; (3) BP ≥130/85 mmHg or antihypertensive treatment; (4) TG ≥1.70 mmol/L or lipid-lowering therapy; (5) HDL-C <1.0 mmol/L (men) or <1.3 mmol/L (women) or lipid-lowering therapy.

### Statistical analysis

We utilized the quartiles of HDL-C, non-HDL-C and NHHR to describe the baseline characteristics of the participants. Continuous variables that follow a normal distribution are shown as the mean (standard deviation), while others are displayed as the median (interquartile range). Categorical variables are represented by numbers and percentages (n(%)). The chi-square test, one-way ANOVA, or the Kruskal-Wallis H test was utilized for group comparisons.

Variables with ≥20% missing data were excluded. For those with <20% missing values, data were assumed missing at random and imputed using multiple imputation methods to create the final dataset. The variance inflation factor was further applied to detect multicollinearity before regression analysis (**Supplementary Tables S1**-**S3**).

The impacts of the quartiles of HDL-C, non-HDL-C and the NHHR and the increase in each standard deviation on MASLD were analyzed via a multivariable Cox regression model. Three models were constructed: Model 1 was built without adjusting for any covariates; Model 2 was adjusted only for sex and age; and Model 3 was further adjusted for BMI, UA, LDL-C, TG, and AST/ALT. The model was adjusted for covariates that resulted in a 10% or greater change in the HR of the predictor variable. The heterogeneity in the associations between NHHR and MASLD within various subgroups was examined using interaction tests. The cumulative incidence rates of MASLD in different quartile groups are presented as Kaplan–Meier curves, and the log-rank test was used to compare the groups. We used restricted cubic spline (RCS) fitting Cox regression models to explore the dose–response relationships between NHHR, HDL-C, non-HDL-C and MASLD after adjusting for the confounding factors as in Model 3, with restricted cubic spline nodes set at the 25th, 50th, 70th, and 95th percentiles of each variable. Once a nonlinear relationship is confirmed, we use a recursive algorithm of the maximum likelihood method to estimate the threshold value. To further investigate the links between NHHR, HDL-C, non-HDL-C, and MASLD, a two-segment Cox proportional hazards model is implemented on both sides of the inflection point. Next, we created a receiver operating characteristic (ROC) curve to evaluate the predictive ability of NHHR, HDL-C, non-HDL-C, and other lipid parameters for new-onset MASLD. The difference in the area under the curve under different parameters was compared via the Delong test.

This study also conducted two sensitivity analyses. To avoid possible causal effects, the first sensitivity analysis excluded the population with a follow-up time of less than 1 year from the study; second, to verify the stability and reliability of the association, the second sensitivity analysis removed any observations with hypercholesteremia (non-HDL-C≥4.1 mmol/L or LDL-C≥3.4 mmol/L) (24). The data were analyzed via Stata 18.0 and R 4.4.1 software, with a *P* value of <0.05 (two-sided) considered statistically significant.

## Results

### Clinical and laboratory variables

The study included 36,897 participants, with an average age of 42.1 years, and 56.5% were women. The occurrence of MASLD among them was 20.3%. **Table 1** provides a summary of the participants’ baseline characteristics according to the NHHR quartiles. The prevalence of MASLD increased with increasing NHHR. Moreover, individuals in these higher-NHHR groups were often male, older, and more prone to diabetes mellitus, hypertension, and hyperuricemia; and have higher measures of BMI, SBP, DBP, WC, Hb, WBC, ALT, AST, GGT, UA, Cr, BUN, FBG, TG, TC, LDL-C, non-HDL-C, and TyG. (all *P* < 0.001). However, there was no statistically significant difference in TBIL levels among the different groups (*P* > 0.05). Similar patterns were noted for the non-HDL-C quartiles (**Supplementary Table S4**). For the HDL-C quartiles, with the exception of the increase in AST/ALT with increasing quartiles, other measures decreased, and the higher HDL-C groups were more likely to be female, younger, and less prone to diabetes, hypertension, and hyperuricemia (all *P* < 0.001) (**Supplementary Table S5**).

**Table 1:** Baseline characteristics of patients based on the quartiles of the NHHR.

Characteristics	All	NHHR quartiles				
		Q1 (<1.87)	Q2 (1.87-2.39)	Q3 (2.4-3.02)	Q4 (>3.02)	*P* value
N	36,897	8,772	9,577	9,581	8,967	
Age, years	42.0 (13.5)	37.8 (12.7)	40.9 (13.2)	43.8 (13.6)	45.4 (13.4)	<0.001
Female, %	20830 (56.5)	6817 (77.7)	6271 (65.5)	4760 (49.7)	2982 (33.3)	<0.001
BMI, kg/m^2^	23.1 (2.7)	21.6 (2.5)	22.6 (2.6)	23.6 (2.6)	24.4 (2.5)	<0.001
SBP, mmHg	124.1 (15.6)	120.2 (14.3)	122.6 (15.3)	125.7 (15.7)	127.7 (16.0)	<0.001
DBP, mmHg	74.6 (10.1)	72.1 (9.3)	73.6 (9.8)	75.5 (10.1)	77.2 (10.3)	<0.001
WC, cm	80.4 (9.0)	75.4 (8.1)	78.6 (8.4)	82.1 (8.3)	85.4 (8.1)	<0.001
PLT, 10^9^/L	239.0 (51.3)	238.5 (50.8)	240.8 (51.7)	238.6 (51.9)	237.9 (50.7)	<0.001
Hb, g/L	141.8(15.5)	135.5 (14.4)	139.1(14.9)	143.7 (15.2)	148.8 (14.3)	<0.001
WBC, 10^9^/L	5.9 (1.5)	5.6 (1.4)	5.8 (1.4)	6.0 (1.4)	6.3 (1.5)	<0.001
ALT, U/L	16.3 (12.5, 22.2)	14.0 (11.0, 18.5)	15.3 (12.0, 20.2)	17.1 (13.2, 23.1)	19.8 (15.0, 26.7)	<0.001
AST, U/L	18.9 (16.1, 22.0)	18.0 (15.6, 21.0)	18.4 (16.0, 21.7)	19.0 (16.5, 22.5)	19.9 (17.0, 23.0)	<0.001
AST/ALT	1.18 (0.36)	1.31 (0.36)	1.24 (0.36)	1.14 (0.34)	1.04 (0.33)	<0.001
ALB, g/L	46.5 (2.5)	46.4 (2.5)	46.4 (2.5)	46.5 (2.6)	46.6 (2.5)	<0.001
GGT, U/L	14.5 (10.8, 21.2)	11.6 (9.1, 15.9)	13.0 (10.0, 18.4)	15.7 (11.7, 22.6)	19.0 (14.0, 28.0)	<0.001
TyG	7.0 (0.5)	6.7 (0.4)	6.8 (0.4)	7.0 (0.4)	7.3 (0.4)	<0.001
TBIL, μmol/L	13.5 (10.8, 17.2)	13.4 (10.8, 17.0)	13.4 (10.8, 17.2)	13.4 (10.8, 17.4)	13.6 (10.8, 17.2)	0.791
DBIL, μmol/L	4.3 (3.4, 5.6)	4.5 (3.5, 5.8)	4.4 (3.4, 5.7)	4.2 (3.3, 5.5)	4.1 (3.3, 5.3)	<0.001
BUN, mmol/L	4.8 (4.0, 5.6)	4.5 (3.8, 5.3)	4.6 (3.9, 5.5)	4.9 (4.1, 5.7)	5.0 (4.2, 5.8)	<0.001
UA, μmol/L	314.7(263.7, 377.0)	279.1 (240.1, 327.8)	299.0 (253.8, 354.5)	327.7 (276.4, 386.1)	361.0 (304.4, 417.9)	<0.001
Cr, μmol/L	63.2 (54.4, 75.6)	58.0 (52.0, 66.4)	60.4 (53.2, 72.3)	65.8 (55.6, 77.0)	71.5 (60.1, 81.0)	<0.001
FPG, mmol/L	5.3 (5.1, 5.7)	5.2 (5.0, 5.5)	5.3 (5.1, 5.6)	5.4 (5.2, 5.7)	5.5 (5.2, 5.8)	<0.001
TC, mmol/L	4.8 (0.8)	4.3 (0.7)	4.6 (0.7)	4.9 (0.8)	5.3 (0.8)	<0.001
TG, mmol/L	1.2 (0.9, 1.6)	0.9 (0.7, 1.2)	1.1 (0.8, 1.4)	1.3 (1.0, 1.6)	1.6 (1.2, 2.1)	<0.001
HDL-C, mmol/L	1.4 (0.3)	1.7 (0.3)	1.5 (0.2)	1.3 (0.2)	1.1 (0.2)	<0.001
LDL-C, mmol/L	2.6 (0.7)	2.0 (0.4)	2.4 (0.5)	2.8 (0.5)	3.2 (0.6)	<0.001
Non-HDL-C, mmol/L	3.4 (0.8)	2.6 (0.5)	3.2 (0.5)	3.6 (0.6)	4.2 (0.6)	<0.001
NHHR	2.4 (1.9, 3.0)	1.6 (1.4, 1.7)	2.1 (2.0, 2.2)	2.7 (2.5, 2.8)	3.6 (3.3, 4.0)	<0.001
Diabetes, %	1324 (3.6)	189 (2.2)	270 (2.8)	387 (4.0)	478 (5.33)	<0.001
Hypertension, %	6804 (18.4)	1135 (12.9)	1534 (16.0)	1928 (20.1)	2207 (24.6)	<0.001
Hyperuricemia, %	6547 (17.7)	821 (9.4)	1231 (12.9)	1908 (19.9)	2587 (28.9)	<0.001
MASLD, %	7487 (20.3)	677 (7.7)	1418 (14.8)	2214 (23.1)	3178 (35.4)	<0.001

Continuous variables are expressed as the mean (standard deviation, SD), median (interquartile range) or n (%). BMI, body mass index; WC, waist circumference; SBP, systolic blood pressure; DBP, diastolic blood pressure; FPG, fasting plasma glucose; TyG, index triglyceride–glucose index; TBil, total bilirubin; DBil, direct bilirubin; ALB, albumin; PLT, platelet; Hb, hemoglobin; WBC, white blood cell; BUN, blood urea nitrogen; TC, total cholesterol; TG, triglyceride; HDL–C, high-density lipoprotein cholesterol; LDL–C, low-density lipoprotein cholesterol; Non-HDL–C, non-high-density lipoprotein cholesterol; ALT, alanine aminotransferase; AST, aspartate transaminase; GGT, gamma–glutamyl transpeptidase; UA, uric acid; Cr, creatinine; NHHR, non-high-density lipoprotein cholesterol to high-density lipoprotein cholesterol ratio; MASLD, metabolic dysfunction-associated steatotic liver disease.

### Associations of HDL-C, non-HDL-C and NHHR with MASLD

**Figure 2:**
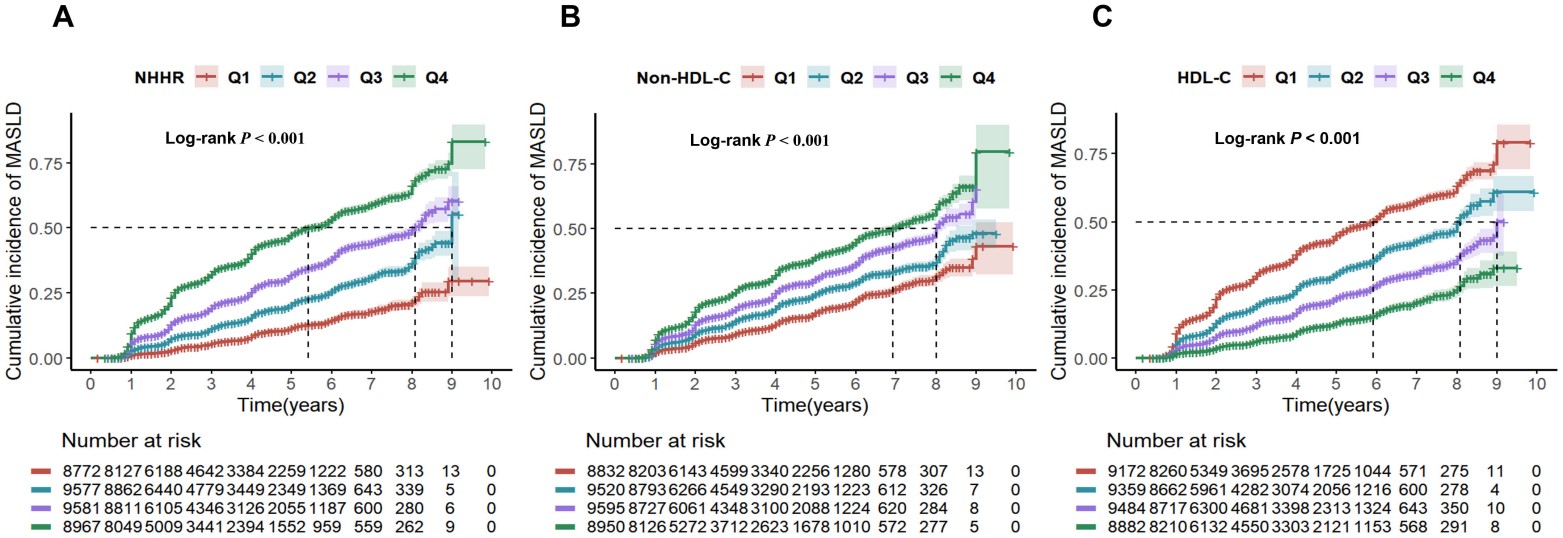
Cumulative risk of MASLD incidence by NHHR **(A)**, non-HDL-C **(B)**, and HDL-C **(C)** quartiles. NHHR, non-high-density lipoprotein cholesterol to high-density lipoprotein cholesterol ratio; Non-HDL-C, non-high-density lipoprotein cholesterol; HDL-C, high-density lipoprotein cholesterol; MASLD, metabolic dysfunction-associated steatotic liver disease.

The patients were followed up for a total of 117,852.67 person-years, with a mean follow-up of 3.19 years. The MASLD incidence per 1,000 person-years rose with non-HDL-C and NHHR quartiles but declined with HDL-C quartiles. With each one standard deviation increase in NHHR, non-HDL-C and HDL-C, the HRs (95% CIs) for the incidence of MASLD were 1.16 (1.12, 1.20), 1.07 (1.05, 1.1) and 0.80 (0.77, 0.82), respectively. We observed a gradual increase in the HR for MASLD as the NHHR and non-HDL-C quartile increased in both the adjusted and unadjusted models, suggesting a positive trend, whereas HDL-C had the opposite trend (all *P* values < 0.001) (**Table 2**). The findings indicated that the NHHR was more closely linked to MASLD in all the models than the non-HDL-C was. Subgroup analysis results are shown in **Supplementary Table S6**. Significant interactions (*P* < 0.05) between baseline NHHR and sex, age, BMI, hypertension, and hyperuricemia suggest these factors notably influenced the NHHR-MASLD risk association across subgroups. The cumulative incidence of MASLD tended to increase as the NHHR, non-HDL-C level groups increased and the HDL-C level group decreased, and the log-rank test revealed significant differences among them (χ²=503.29, *P* < 0.001). (**Figure 2**).

**Table 2:** Relationships between HDL-C, non-HDL-C, NHHR and MASLD.

Variables	Per 1000 person-year	Model 1 HR (95%CI), *P* value	Model 2 HR (95%Cl), *P* value	Model 3 HR (95%CI), *P* value
NHHR				
Per SD increase		1.71(1.68, 1.75) <0.001	1.57(1.53, 1.60) <0.001	1.16(1.12, 1.20) <0.001
Q1 (<1.87)	22.46	Reference	Reference	Reference
Q2 (1.87-2.39)	44.43	1.98(1.83, 2.15) <0.001	1.83(1.69, 1.99) <0.001	1.49(1.37, 1.62) <0.001
Q3 (2.40-3.02)	73.52	3.36(3.11, 3.63) <0.001	2.80(2.58, 3.02) <0.001	1.87(1.72, 2.04) <0.001
Q4 (>3.02)	123.74	5.90(5.48, 6.36) <0.001	4.45(4.13, 4.81) <0.001	2.34(2.13, 2.56) <0.001
*P* for trend		<0.001	<0.001	<0.001
Non-HDL-C				
Per SD increase		1.42(1.39, 1.45) <0.001	1.35(1.32, 1.38) <0.001	1.07(1.05, 1.1) <0.001
Q1 (<2.81)	36.90	Reference	Reference	Reference
Q2 (2.81-3.33)	53.27	1.47(1.37, 1.58) <0.001	1.36(1.27, 1.46) <0.001	1.11(1.03, 1.20) <0.001
Q3 (3.34-3.91)	71.88	2.03(1.90, 2.17) <0.001	1.75(1.64, 1.87) <0.001	1.14(1.06, 1.23) <0.001
Q4 (>3.91)	95.95	2.81(2.64, 3.00) <0.001	2.34(2.19, 2.50) <0.001	1.22(1.13, 1.31) <0.001
*P* for trend		<0.001	<0.001	<0.001
HDL-C				
Per SD increase		0.54(0.53, 0.56) <0.001	0.61(0.59, 0.62) <0.001	0.80(0.77, 0.82) <0.001
Q1 (<1.20)	115.65	Reference	Reference	Reference
Q2 (1.20-1.39)	72.04	0.59(0.56, 0.62) <0.001	0.67(0.63, 0.70) <0.001	0.92(0.87, 0.97) 0.004
Q3 (1.40-1.61)	46.78	0.38(0.36, 0.40) <0.001	0.47(0.44, 0.50) <0.001	0.76(0.71, 0.81) <0.001
Q4 (>1.61)	25.53	0.21(0.20, 0.23) <0.001	0.20(0.26, 0.31) <0.001	0.55(0.50, 0.60) <0.001
*P* for trend		<0.001	<0.001	<0.001

Model 1: no covariates were adjusted. Model 2: Age and sex were adjusted. Model 3: Sex, age, BMI, UA, LDL-C, TG and AST/ALT were adjusted for. LDL-C could not be included in the same model because of the collinearity between LDL-C and non-HDL-C. HR, hazard ratio; CI, confidence interval; BMI, body mass index; TG, triglyceride; ALT, alanine aminotransferase; AST, aspartate aminotransferase; UA, uric acid; HDL-C, high-density lipoprotein cholesterol; LDL-C, low-density lipoprotein cholesterol; Non-HDL-C, non-high-density lipoprotein cholesterol; NHHR, non-high-density lipoprotein cholesterol to high-density lipoprotein cholesterol ratio; MASLD, metabolic dysfunction-associated steatotic liver disease.

### Dose–response relationship between the NHHR and risk of MASLD

**Figure 3:**
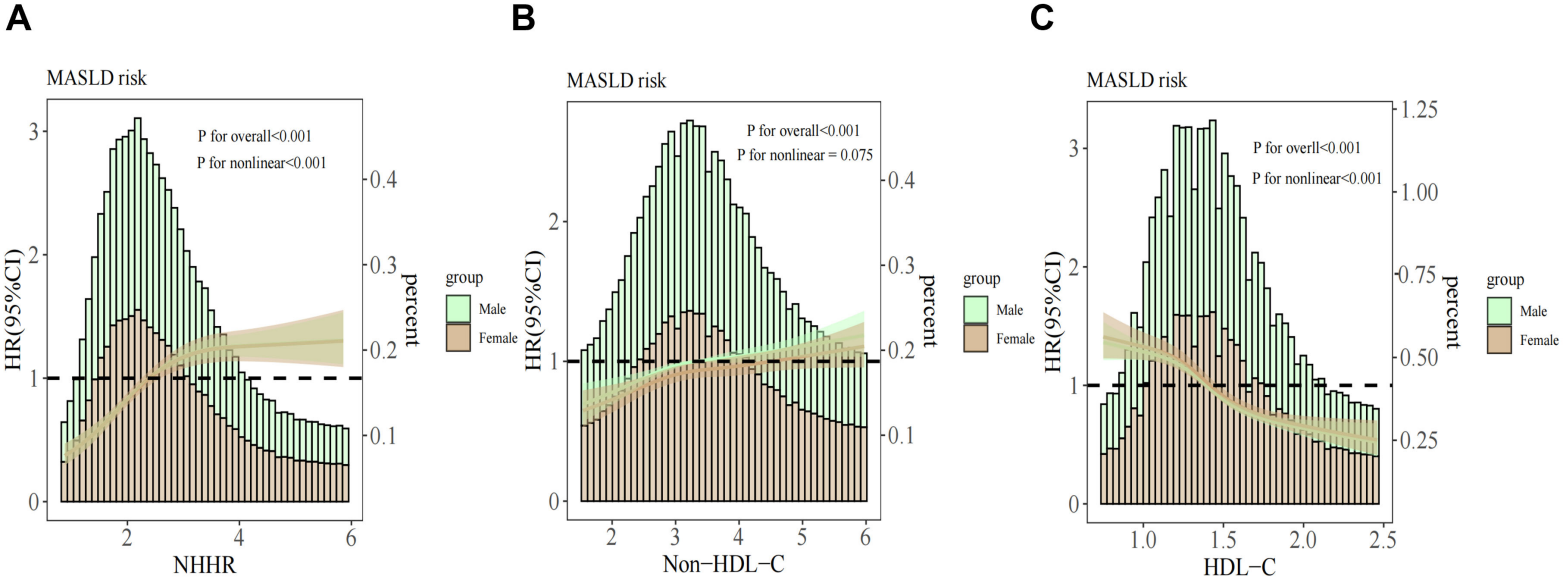
Association between the NHHR **(A)**, non-HDL-C **(B)**, HDL-C **(C)** and MASLD stratified by sex. The covariates adjusted to the model were the same as those previously described. NHHR, non-high-density lipoprotein cholesterol to high-density lipoprotein cholesterol ratio; Non-HDL-C, non-high-density lipoprotein cholesterol; HDL-C, high-density lipoprotein cholesterol; MASLD, metabolic dysfunction-associated steatotic liver disease; HR, hazard ratio; CI, confidence interval.

Dose-response relationships between NHHR, HDL-C, non-HDL-C, and MASLD risk were analyzed using RCS, with adjustments for age, sex, BMI, UA, LDL-C, TG, and AST/ALT. Results (**Figure 3**) revealed a nonlinear relationship for NHHR and HDL-C (*P* for nonlinearity <0.001) and a positive linear association for non-HDL-C (*P* for nonlinearity = 0.075). Additionally, the infection points for MASLD of NHHR and HDL-C were identified as 2.54 and 1.31, respectively (both *P* values for log-likelihood ratio < 0.05) (**Table 3**). After adjusting for various factors, each unit increase in the NHHR below the threshold increased the risk of MASLD by 81% (HR: 1.81, 95% CI: 1.64–1.99, *P*<0.001). Above the threshold, a unit increase in the NHHR increased the risk by 8% (HR: 1.08, 95% CI: 1.03–1.13, *P* = 0.001). For HDL-C, on the left side of the infection point, the HR was 0.60 (95% CI: 0.50–0.73); on the right side of the infection point, the HR was 0.40 (95% CI: 0.34–0.47). In both cases, *P* < 0.001. The stratified analyses revealed a greater risk of MASLD under equivalent non-HDL-C concentrations among males.

**Table 3:** Threshold effect analysis of the effects of the NHHR and HDL-C level on the risk of MASLD.

Variables	HR (95%CI)	*P* value
NHHR		
Fitting by the standard Cox proportional risk model	1.19 (1.15, 1.24)	<0.001
Fitting by the two-piecewise Cox proportional risk model		
Inflection point	2.54	
NHHR < 2.54	1.81 (1.64, 1.99)	<0.001
NHHR ≥ 2.54	1.08 (1.03, 1.13)	0.001
*P* for Log-likelihood ratio	<0.001	
HDL-C		
Fitting by the standard Cox proportional risk model	0.48 (0.43, 0.53)	<0.001
Fitting by the two-piecewise Cox proportional risk model		
Inflection point	1.31	
HDL-C < 1.31	0.60 (0.50, 0.73)	<0.001
HDL-C ≥ 1.31	0.40 (0.34, 0.47)	<0.001
*P* for Log-likelihood ratio	0.007	

Adjusted for sex, age, BMI, UA, LDL-C, TG and AST/ALT. HR, hazard ratio; CI, confidence interval; BMI, body mass index; TG, triglyceride; ALT, alanine aminotransferase; AST, aspartate aminotransferase; UA, uric acid; HDL-C, high-density lipoprotein cholesterol; LDL-C, low-density lipoprotein cholesterol; NHHR, non-high-density lipoprotein cholesterol to high-density lipoprotein cholesterol ratio; MASLD, metabolic dysfunction-associated steatotic liver disease.

### Sensitivity analyses

**Figure 4:**
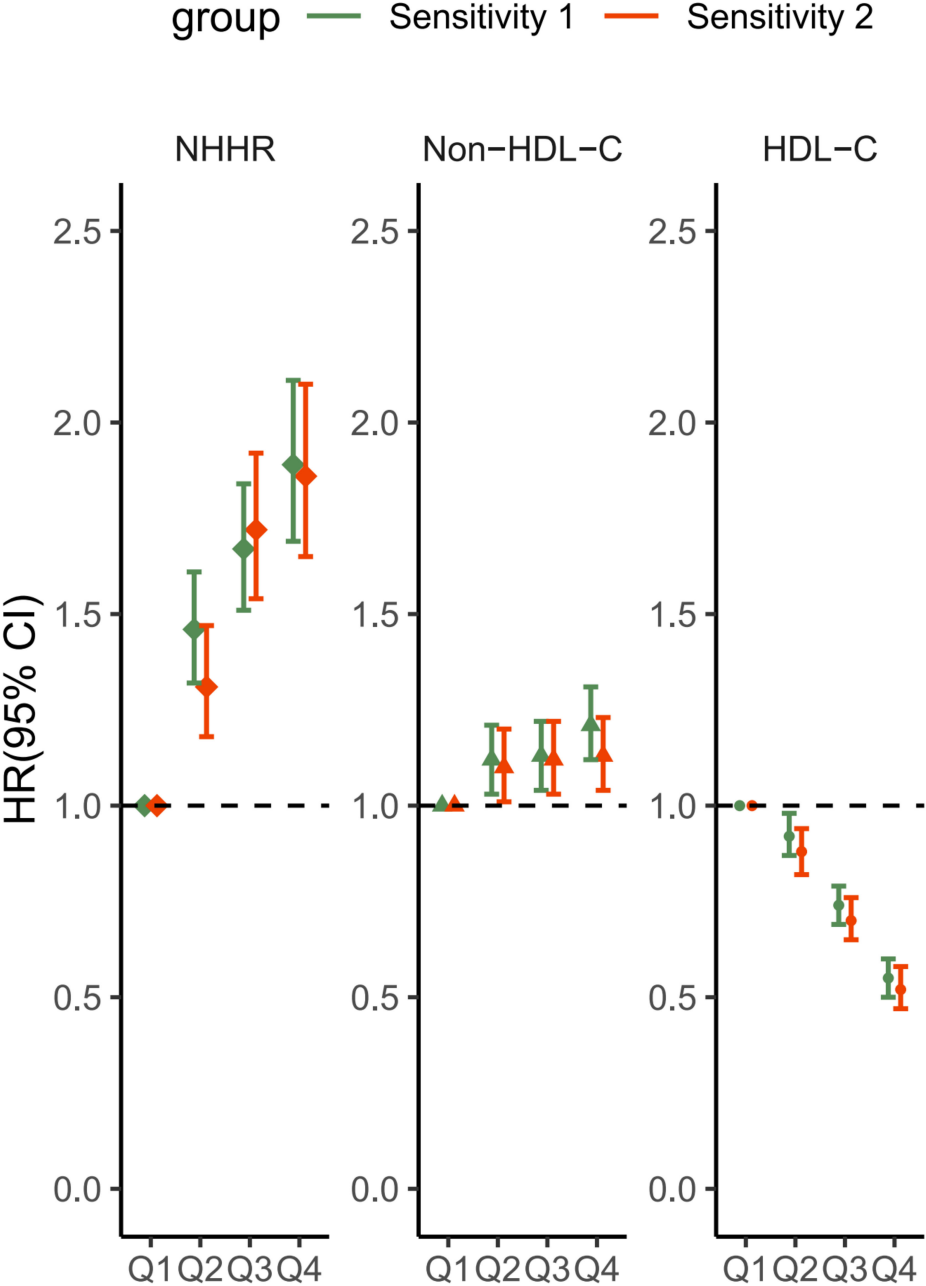
Sensitivity analysis of the associations between HDL-C, non-HDL-C and the NHHR with the MASLD score. Sensitivity 1: Patients with a follow-up time of less than 1 year were excluded. Sensitivity 2: Subjects with hypercholesterolemia were excluded. The covariates adjusted to the model were the same as those previously described. NHHR, non-high-density lipoprotein cholesterol to high-density lipoprotein cholesterol ratio; Non-HDL-C, non-high-density lipoprotein cholesterol; HDL-C, high-density lipoprotein cholesterol; MASLD, metabolic dysfunction-associated steatotic liver disease; HR, hazard ratio; CI, confidence interval.

The sensitivity analysis outcomes were shown in **Figure 4**. Upon excluding the participants during the initial one-year period of follow-up, a total of 33,850 cases remained; the second sensitivity analysis removed any observations with hypercholesteremia, and 29,548 participants remained. After adjusting for confounders, the estimated effects of the associations of HDL-C, non-HDL-C and NHHR with MASLD remained virtually unchanged.

### ROC analysis

**Figure 5:**
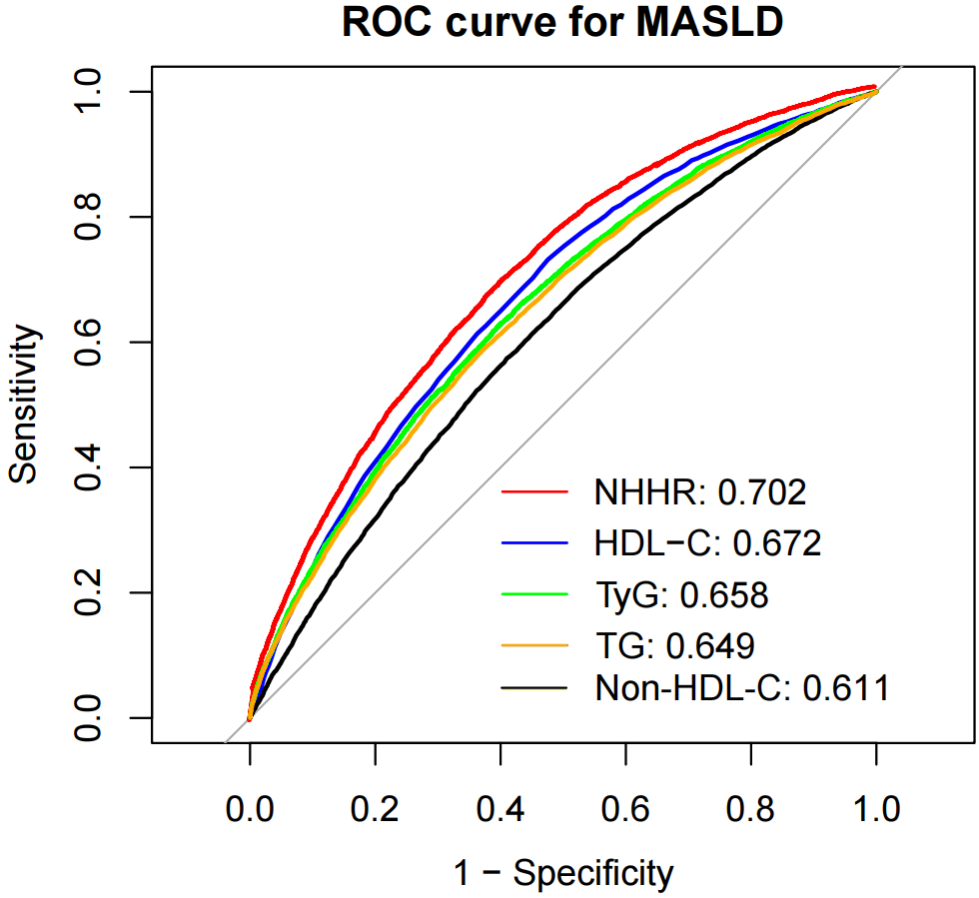
Receiver operating characteristic curve analysis of MASLD-related lipid parameters. NHHR, non-high-density lipoprotein cholesterol to high-density lipoprotein cholesterol ratio; Non-HDL-C, non-high-density lipoprotein cholesterol; HDL-C, high-density lipoprotein cholesterol; TyG, index triglyceride–glucose index; TG, triglyceride; MASLD, metabolic dysfunction-associated steatotic liver disease; HR, hazard ratio; CI, confidence interval.

According to the ROC curve results, the areas under the curve (AUCs) for the NHHR, HDL-C, TyG, TG, and non-HDL-C were 0.702, 0.672, 0.658, 0.649 and 0.611, respectively (**Figure 5**). Among them, the AUC of the NHHR was the largest, with a sensitivity of 0.714 and specificity of 0.597 (**Supplementary Table S7**), suggesting that the NHHR may be a better indicator of MASLD risk assessment (all DeLong *P* < 0.05). The best cutoffs for the NHHR, non-HDL-C and HDL-C were 2.536, 3.210 and 1.410, respectively, with Youden indices of 0.279, 0.164, and 0.256, respectively. Time-dependent ROC analysis was carried out to evaluate the predictive capacity of the NHHR in the model for MASLD. The outcomes demonstrated that the AUCs of the model’s predictive values for all-cause mortality at 3, 5 and 8 years were 0.716, 0.694 and 0.699, respectively. These results suggest that the model seems to possess effective predictive value for MASLD in both the short and long term (**Supplementary Figure S1**).

## Discussion

In this population-based perspective cohort study, we examined the associations among non-HDL-C, HDL-C, the NHHR and MASLD. A higher NHHR is strongly associated with a greater risk of developing MASLD, and the NHHR was better at recognizing MASLD than other lipid indicators alone. This relationship remained consistent across various subgroups and sensitivity analyses. Additionally, the cutoff values for the detection of MASLD were 1.410 for HDL-C, 3.210 for non-HDL-C, and 2.536 for the NHHR.

In most of the previous studies from China, the prevalence of MASLD among the general population ranged from 9.71% to 39.27%, whereas our prevalence was 20.3% (19, 25, 26). MASLD stems from hepatic lipid overload, driven by upregulated *de novo* lipogenesis and impaired fatty acid clearance (27). Recent studies have concluded that MASLD at all stages is linked to increased cardiovascular event risk, independent of metabolic syndrome factors, and that fibrosis worsens this risk (28, 29). The main feature of dyslipidemia in patients with MASLD is an atherogenic lipid profile, including high TG levels, increased concentrations of small dense LDL particles and low HDL-C levels (30). The alterations in hepatic lipid metabolism that lead to MASLD also cause atherosclerotic dyslipidemia, particularly elevated TG and RC, and the infiltration of small dense LDL particles into the arterial wall, promoting the formation of atherosclerotic plaques (31). This may be why these patients have a greater chance of developing CVD. MASLD shares many risk factors with CVD, including obesity, insulin resistance, type 2 diabetes, and atherogenic dyslipidemia. Therefore, some of the risk factors used to predict CVD have also been shown to have good predictive value for MASLD.

LDL-C is a well-known causal risk factor for atherosclerosis and CVD (32). However, LDL cholesterol levels alone may underestimate the true cholesterol burden in MASLD patients, as they do not fully capture lipoprotein particle quality. Non-HDL-C is a composite measure that includes the mass of cholesterol in all atherogenic conditions and reflects the extent of coronary artery damage; it is a more detailed marker for atherosclerosis than one type of lipoprotein cholesterol alone (33). Several previous studies have also suggested that non-HDL-C levels are an important risk factor for MASLD (6, 34, 35). In our study, the RCS analysis revealed that there was no significant nonlinear association between non-HDL-C and the risk of developing MASLD. Moreover, men had a higher MASLD risk at the same non-HDL-C level. Some studies have also shown that in the same non-HDL-C concentration range, men have a greater risk of CVD events than women do (36, 37). This may be due to estrogen-induced cholesterol reduction and blood vessel protection in premenopausal women (38). In addition, our study revealed that TG had a greater AUC than non-HDL-C for the detection of MASLD, and the optimal cutoff value of 3.210 mmol/L for non-HDL-C in identifying individuals with MASLD may be useful in screening high-risk groups with MASLD. In conclusion, the detection and treatment of dyslipidemia via non-HDL-C is still important in patients with MASLD. HDL-C particles may be associated with proteins with anti-inflammatory and antioxidant functions (39). Recent research has revealed a U-shaped curve between patients’ HDL-C levels and all-cause mortality, cardiovascular mortality or stroke risk (10, 40), so it may be time to decrease the level of good cholesterol. In MASLD, the lower number of HDL-C particles observed in patients with MASLD may impair cholesterol homeostasis, and MASLD has been shown to be associated with an altered HDL proteome (41). More importantly, a complex set of changes in LDL-C and HDL-C is thought to be the primary cause of increased cardiovascular risk in patients with MASLD (42). Previous studies have verified the correlation between low serum HDL-C levels and MASLD occurrence in various populations (43, 44). A previous study reported a saturation effect on the link between HDL-C and MASLD onset; when HDL-C > 2.19 mmol/L, this protective relationship disappears, which also indicates that higher HDL-C is not always good (44). We also found that higher quartiles of HDL-C were associated with a lower incidence of MASLD in adults, with sensitivity analyses further confirming the relationship’s stability. Further analysis revealed a nonlinear negative relationship between serum HDL-C levels and MASLD risk, yet no saturation effect was detected. Notably, in the ROC analysis, the optimal threshold for HDL-C to predict MASLD risk was 1.410 mmol/L. The association between baseline lipid profiles and MASLD has been further extended to longitudinal changes in TG and HDL-C, which correlate independently with MASLD (45). Overall, further research is needed to better understand the link between HDL-C function and MASLD and its significance for disease progression. The NHHR was first used to assess the risk of atherosclerosis (46). Wang et al. first demonstrated that the NHHR is an independent predictor of MASLD and is more useful than non-HDL-C (25). Our study revealed that in all the models, compared with non-HDL-C, an increase in the NHHR was more significantly associated with an increased risk of MASLD. Furthermore, the subgroup analysis revealed that sex, age, BMI, hypertension, and hyperuricemia were significantly associated with the NHHR (*P* for interaction < 0.05). As the level of NHHR exposure increased, the risk of MASLD increased more significantly in low-risk groups, including females, younger age groups, those with a BMI < 25 kg/m², nonhypertensive individuals, and nonhyperuricemic individuals. The mean age of the female participants in the study was 40.1 ± 12.6 years, which was below the typical age of menopause. Consequently, estrogen levels were higher than those observed postmenopause. Research has demonstrated that estrogen deficiency can exacerbate nonalcoholic steatohepatitis (NASH) in a mouse model of MASLD (47). NHHR’s sensitivity in low-risk populations may arise from heightened metabolic fragility to lipid imbalance, sex-specific hormonal modulation of lipoprotein dynamics, and overlooked subclinical metabolic tipping points. The NHHR was associated with a more significant increase in the risk of MASLD in those without hypertension and hyperuricemia, likely reflecting enhanced biomarker sensitivity to occult metabolic dysfunction in healthier cohorts. In populations with hypertension and hyperuricemia, the impact of the NHHR on the risk of MASLD may be obscured by the use of medications, therapeutic interventions, the presence of comorbidities and lifestyle choices. Therefore, these potential influencing factors should be accounted for in clinical practice. In addition, the RCS analysis revealed a nonlinear positive association between the NHHR and MASLD risk after adjustment for confounders. At NHHR=2.54, MASLD risk stabilized (HR approaching 1 at NHHR=2.39), aligning with a NHANES cross-sectional study, whereas a separate NHANES analysis revealed an S-shaped pattern where MASLD probability decreased with rising NHHR below the threshold (18, 48). Moreover, recent NHHR-MASLD cohort studies have revealed a saturation effect or downward trend after the inflection point, and the inflection points are 3.5 and 3.34, respectively (25, 26). These findings indicate that the MASLD risk does not increase when the NHHR exceeds a certain value. Our threshold of 2.54 differs from that of the above study. This may be because our study uses the two-piecewise Cox proportional risk model, whereas their study uses the two-piecewise linear regression model, and we have a larger sample size. Similarly, research by Xuan et al. revealed that elevated NHHR is independently associated with an increased risk of MASLD and liver fibrosis and is a better predictor of MASLD than non-HDL-C or HDL-C alone (19). Our study revealed that, compared with HDL-C, TyG, TG and non-HDL-C measurements, the NHHR has a superior diagnostic predictive value for the risk of developing MASLD, and an NHHR value of 2.536 may be a potential intervention threshold for MASLD risk. Higher NHHR levels are associated with increased non-HDL-C—particularly LDL-C and very-low-density lipoprotein cholesterol (VLDL-C)—and decreased HDL-C. These elevated LDL and VLDL levels raise the risk of fat accumulation in the liver by directly promoting the buildup of cholesterol and triglycerides. The ectopic accumulation of lipids in hepatocytes generates reactive oxygen species, which lead to lipid peroxidation, a state of oxidative stress and the release of several cytokines (49). The inflammatory conditions described above lead to hepatocellular apoptosis, the deposition of collagen and the abnormal proliferation of surviving hepatocytes, resulting in chronic liver cell damage, cirrhosis and ultimately liver cancer (50). In contrast, HDL-C exerts protective effects by preventing hepatic lipid accumulation via reverse cholesterol transport, antioxidant, and anti-inflammatory mechanisms (51). This likely explains the link between higher HDL-C levels and reduced MASLD risk in our study, as well as the strong association between NHHR and MASLD.

In our long-term study with a large sample, we analyzed HDL-C, non-HDL-C and NHHR in relation to MASLD to make them more comparable. We further confirmed that the NHHR is a novel composite indicator better at predicting MASLD than other lipid indicators in our study. Further sensitivity analysis suggested good stability in the independent correlations between NHHR, HDL-C, and non-HDL-C and MASLD. There are several limitations. First, While liver biopsy remains the gold standard for diagnosing MASLD, our study employed liver ultrasonography as the primary diagnostic tool, potentially missing early-stage steatosis. Second, this study focused only on baseline NHHR values and lacked analysis of longitudinal NHHR changes and their association with MASLD progression. Third, potential selection bias and unmeasured confounding inherent to retrospective designs, compounded by the lack of external validation limiting generalizability, require verification through multi-center prospective cohorts.

## Conclusion

This study demonstrates that elevated NHHR and non-HDL-C levels predict increased MASLD risk, while higher HDL-C inversely correlates with disease prevalence. MASLD risk is strongly linked to NHHR < 2.54. It rises more notably in females, younger groups, those with BMI < 25 kg/m², nonhypertensive and nonhyperuricemic individuals. Also, elevated non-HDL-C raises MASLD risk more in men than in women. This underscores the need for sex-specific and metabolic profile-tailored screening strategies. This study suggests that the NHHR may be a valuable, novel predictor of MASLD, with greater predictive power than other lipid parameters. Clinicians should integrate NHHR into risk stratification protocols to enhance MASLD detection, while researchers must address current evidence gaps through multicenter validation cohorts.

## Data Availability

The raw data supporting the conclusions of this article will be made available by the authors, without undue reservation.
