# Supplementary material for: Associations between high-density lipoprotein cholesterol, non-high-density lipoprotein cholesterol, and their ratio with metabolic dysfunction-associated steatotic liver disease: a retrospective cohort study
Source: Front Endocrinol (Lausanne). 2025 Jun 18;16:1585811. doi: 10.3389/fendo.2025.1585811 (PMC12213369; doi:10.3389/fendo.2025.1585811)
Supplement: Supplementary file 1 [file DataSheet1.docx]

**The Association of HDL-C, non-HDL-C and Their Ratio (NHHR) with Metabolic Dysfunction-Associated Steatotic Liver Disease**

**Supplementary Material**

**Table S1.** Collinearity diagnostics of HDL-C with other covariates.

**Table S2.** Collinearity diagnostics of non-HDL-C with other covariates.

**Table S3.** Collinearity diagnostics of NHHR with other covariates.

**Table S4.** Baseline characteristics of patients based on the quartiles of non-HDL-C.

**Table S5.** Baseline characteristics of patients based on the quartiles of HDL-C.

**Table S6.** Association between NHHR and MASLD in subgroups.

**Table S7.** Areas under the receiver operating characteristic curves for each lipid parameter in identifying MASLD.

**Figure S1** Time-dependent ROC curves for the prediction of MASLD.

**Table S1** Collinearity diagnostics of HDL-C with other covariates

| Variables | Collinearity Statistics | |
| --- | --- | --- |
|  | Tolerance | Variance inflation factor |
| HDL-C | 0.40 | 1.32 |
| Sex | 0.61 | 1.67 |
| Age (year) | 0.88 | 1.14 |
| BMI (kg/m^2^) | 0.83 | 1.22 |
| UA (μmol/L) | 0.60 | 1.65 |
| TG (mmol/L) | 0.67 | 1.21 |
| LDL-C (mmol/L) | 0.55 | 1.09 |
| AST/ ALT | 0.81 | 1.23 |

HDL-C, high-density lipoprotein cholesterol; BMI, body mass index; UA, uric acid;TG, triglyceride; LDL-C, low-density lipoprotein cholesterol; ALT, Alanine aminotransferase; AST, aspartate transaminase.

**Table S2** Collinearity diagnostics of non-HDL-C with other covariates

| Variables | Collinearity Statistics | |
| --- | --- | --- |
|  | Tolerance | Variance inflation factor |
| Non-HDL-C (mmol/L) | 0.12 | 8.03 |
| Sex | 0.62 | 1.61 |
| Age (year) | 0.88 | 1.13 |
| BMI (kg/m^2^) | 0.84 | 1.19 |
| UA (μmol/L) | 0.60 | 1.66 |
| TG (mmol/L) | 0.59 | 1.69 |
| LDL-C (mmol/L) | 0.14 | 7.12 |
| AST/ ALT | 0.82 | 1.22 |

Non-HDL-C, non-high-density lipoprotein cholesterol; BMI, body mass index; UA, uric acid;TG, triglyceride; LDL-C, low-density lipoprotein cholesterol; ALT, Alanine aminotransferase; AST, aspartate transaminase.

**Table S3** Collinearity diagnostics of NHHR with other covariates

| Variables | Collinearity Statistics | |
| --- | --- | --- |
|  | Tolerance | Variance inflation factor |
| NHHR | 0.40 | 2.52 |
| Sex | 0.61 | 1.65 |
| Age (year) | 0.88 | 1.14 |
| BMI (kg/m^2^) | 0.83 | 1.21 |
| UA (μmol/L) | 0.60 | 1.66 |
| TG (mmol/L) | 0.67 | 1.50 |
| LDL-C (mmol/L) | 0.55 | 1.82 |
| AST/ ALT | 0.81 | 1.23 |

NHHR, non-high-density lipoprotein cholesterol to high-density lipoprotein cholesterol ratio; BMI, body mass index; UA, uric acid;TG, triglyceride; LDL-C, low-density lipoprotein cholesterol; ALT, Alanine aminotransferase; AST, aspartate transaminase.

**Table S4** Baseline characteristics of patients based on the quartiles of non-HDL-C

| **Characteristics** | **Non-HDL-C quartiles** |  |  |  |  |
| --- | --- | --- | --- | --- | --- |
|  | **Q1(<2.81)** | **Q2(2.81–3.33)** | **Q3(3.34–3.91)** | **Q4(>3.91)** | ***P*-value** |
| **N** | 8,832 | 9,520 | 9,595 | 8,950 |  |
| **Age, years** | 36.9 (12.3) | 40.1 (12.6) | 43.6 (13.4) | 47.6 (13.3) | <0.001 |
| **Female, %** | 5925 (67.1%) | 5556 (58.4%) | 4966 (51.8%) | 4383 (49.0%) | <0.001 |
| **BMI, kg/m^2^** | 22.1 (2.7) | 22.8 (2.7) | 23.4 (2.7) | 23.8 (2.6) | <0.001 |
| **SBP, mmHg** | 120.5 (14.3) | 122.6 (15.0) | 125.1 (15.6) | 128.0 (16.5) | <0.001 |
| **DBP, mmHg** | 72.3 (9.4) | 73.9 (9.8) | 75.3 (10.0) | 76.9 (10.5) | <0.001 |
| **WC, cm** | 77.1 (8.7) | 79.5 (8.9) | 81.6 (8.9) | 83.3 (8.5) | <0.001 |
| **PLT, 10^9^/L** | 237.3 (51.4) | 238.8 (51.6) | 238.3 (51.6) | 241.6 (50.4) | <0.001 |
| **Hb, g/L** | 137.9 (15.8) | 140.7 (15.6) | 143.1 (15.3) | 145.4 (14.4) | <0.001 |
| **WBC, 10^9^/L** | 5.8 (1.4) | 5.8 (1.4) | 6.0 (1.5) | 6.0 (1.5) | <0.001 |
| **ALT, U/L** | 14.4 (11.2,19.3) | 15.8 (12.0,21.1) | 17.0 (13.0,23.0) | 18.6 (14.2,25.0) | <0.001 |
| **AST, U/L** | 17.9 (15.5,21.0) | 18.5 (16.0,21.7) | 19.0 (16.4,22.4) | 20.0 (17.0,23.1) | <0.001 |
| **AST/ALT** | 1.27 (0.37) | 1.21 (0.36) | 1.16 (0.36) | 1.10 (0.34) | <0.001 |
| **ALB, g/L** | 46.4 (2.5) | 46.5 (2.5) | 46.5 (2.6) | 46.5 (2.5) | <0.001 |
| **GGT, U/L** | 12.0 (9.5,16.7) | 13.6 (10.3,19.3) | 15.4 (11.3,22.4) | 18.0 (13.0,27.0) | <0.001 |
| **TyG** | 6.7 (0.4) | 6.9 (0.4) | 7.0 (0.4) | 7.2 (0.4) | <0.001 |
| **TBIL, μmol/L** | 13.5 (10.7,17.3) | 13.4 (10.8,17.3) | 13.5 (10.8,17.3) | 13.5 (10.8,17.0) | 0.890 |
| **DBIL, μmol/L** | 4.7 (3.7,6.1) | 4.4 (3.5,5.7) | 4.2 (3.4,5.4) | 3.9 (3.1,4.9) | <0.001 |
| **BUN, mmol/L** | 4.5 (3.8,5.3) | 4.7 (3.9,5.5) | 4.8 (4.1,5.7) | 5.0 (4.3,5.8) | <0.001 |
| **UA, μmol/L** | 292.3 (247.6,348.1) | 307.4 (258.5,368.4) | 323.0 (271.1,386.1) | 337.0 (281.4,399.2) | <0.001 |
| **SCr, μmol/L** | 59.2 (52.5,70.7) | 62.2 (54.0,74.9) | 65.0 (55.4,77.0) | 67.0 (56.9,78.1) | <0.001 |
| **FPG, mmol/L** | 5.2 (5.0,5.5) | 5.3 (5.1,5.6) | 5.4 (5.1,5.7) | 5.5 (5.2,5.8) | <0.001 |
| **TC, mmol/L** | 3.9 (0.4) | 4.5 (0.3) | 5.0 (0.4) | 5.9 (0.5) | <0.001 |
| **TG, mmol/L** | 0.9 (0.7,1.2) | 1.1 (0.8,1.5) | 1.3 (0.9,1.7) | 1.5 (1.1,2.0) | <0.001 |
| **HDL-C, mmol/L** | 1.5 (0.3) | 1.4 (0.3) | 1.4 (0.3) | 1.4 (0.3) | <0.001 |
| **LDL-C, mmol/L** | 1.8 (0.3) | 2.3 (0.2) | 2.8 (0.3) | 3.4 (0.4) | <0.001 |
| **Non-HDL-C, mmol/L** | 2.4 (0.3) | 3.1 (0.2) | 3.6 (0.2) | 4.5 (0.4) | <0.001 |
| **NHHR** | 1.7 (1.4,2.0) | 2.2 (1.9,2.5) | 2.6 (2.2,3.1) | 3.3 (2.8,3.9) | <0.001 |
| **Diabetes, %** | 251 (2.8%) | 244 (2.6%) | 401 (4.2%) | 428 (4.8%) | <0.001 |
| **Hypertension, %** | 1126 (12.7%) | 1470 (15.4%) | 1939 (20.2%) | 2269 (25.4%) | <0.001 |
| **Hyperuricemia, %** | 1029 (11.7%) | 1445 (15.2%) | 1923 (20.0%) | 2150 (24.0%) | <0.001 |
| **MASLD, %** | 1110 (12.6%) | 1646 (17.3%) | 2167 (22.6%) | 2564 (28.6%) | <0.001 |

Continuous variables are expressed as mean (standard deviation, SD), median (interquartile range) or n (%). BMI, body mass index; WC, waist circumference; SBP, systolic blood pressure; DBP, diastolic blood pressure; FPG, fasting plasma glucose; TyG, index triglyceride-glucose index; TBil, total bilirubin; DBil, direct Bilirubin; ALB, albumin; PLT, platelet; Hb, hemoglobin; WBC, white blood cell; BUN, blood urea nitrogen; TC, total cholesterol; TG, triglyceride; HDL-C, high-density lipoprotein cholesterol; LDL-C, low-density lipoprotein cholesterol; Non-HDL-C, non-high-density lipoprotein cholesterol; ALT, Alanine aminotransferase; AST, aspartate transaminase; GGT, gamma-glutamyl transpeptidase; UA, uric acid; Cr, creatinine; NHHR, non-high-density lipoprotein cholesterol to high-density lipoprotein cholesterol ratio; MASLD, metabolic dysfunction-associated steatotic liver disease.

**Table S5** Baseline characteristics of patients based on the quartiles of HDL-C

| **Characteristics** | **HDL-C quartiles** |  |  |  |  |
| --- | --- | --- | --- | --- | --- |
|  | **Q1(<1.20)** | **Q2(1.20–1.39)** | **Q3(1.40–1.61)** | **Q4(>1.61)** | ***P*-value** |
| **N** | 9,172 | 9,359 | 9,484 | 8,882 |  |
| **Age, years** | 42.5 (13.3) | 41.9 (13.4) | 41.6 (13.5) | 42.2 (13.8) | <0.001 |
| **Female, %** | 2707 (29.5%) | 4748 (50.7%) | 6272 (66.1%) | 7103 (80.0%) | <0.001 |
| **BMI, kg/m^2^** | 24.3 (2.6) | 23.4 (2.6) | 22.7 (2.6) | 21.9 (2.5) | <0.001 |
| **SBP, mmHg** | 126.1 (15.4) | 124.4 (15.7) | 123.2 (15.5) | 122.4 (15.8) | <0.001 |
| **DBP, mmHg** | 76.2 (10.2) | 75.0 (10.0) | 73.9 (10.0) | 73.2 (9.7) | <0.001 |
| **WC, cm** | 85.1 (8.3) | 81.5 (8.6) | 78.9 (8.5) | 76.1 (8.2) | <0.001 |
| **PLT, 10^9^/L** | 234.8 (51.3) | 239.7 (51.8) | 241.1 (51.3) | 240.4 (50.5) | <0.001 |
| **Hb, g/L** | 148.7 (15.1) | 143.2 (15.6) | 139.3 (15.0) | 135.8 (13.4) | <0.001 |
| **WBC, 10^9^/L** | 6.3 (1.4) | 6.0 (1.4) | 5.8 (1.4) | 5.5 (1.4) | <0.001 |
| **ALT, U/L** | 19.0 (14.4,26.0) | 16.7 (12.8,23.0) | 15.6 (12.0,20.7) | 14.8 (11.7,19.2) | <0.001 |
| **AST, U/L** | 19.1 (16.6,22.6) | 18.7 (16.0,22.0) | 18.5 (16.0,21.9) | 18.9 (16.1,22.0) | <0.001 |
| **AST/ALT** | 1.05 (0.33) | 1.15 (0.35) | 1.23 (0.36) | 1.30 (0.36) | <0.001 |
| **ALB, g/L** | 46.6 (2.6) | 46.5 (2.6) | 46.4 (2.5) | 46.4 (2.5) | <0.001 |
| **GGT, U/L** | 18.0 (13.0,25.6) | 15.0 (11.0,22.0) | 13.4 (10.1,19.6) | 12.3 (9.7,17.2) | <0.001 |
| **TyG** | 7.2 (0.5) | 7.0 (0.4) | 6.9 (0.4) | 6.8 (0.4) | <0.001 |
| **TBIL, μmol/L** | 13.6 (10.7,17.6) | 13.4 (10.8,17.3) | 13.4 (10.8,17.0) | 13.4 (10.9,16.9) | 0.209 |
| **DBIL, μmol/L** | 4.5 (3.5,5.8) | 4.4 (3.4,5.7) | 4.3 (3.4,5.5) | 4.1 (3.3,5.3) | <0.001 |
| **BUN, mmol/L** | 4.9 (4.2,5.7) | 4.8 (4.0,5.6) | 4.7 (4.0,5.5) | 4.7 (4.0,5.5) | <0.001 |
| **UA, μmol/L** | 359.0 (303.4,416.0) | 325.7 (273.3,385.0) | 299.8 (255.0,356.1) | 280.8 (242.7,330.1) | <0.001 |
| **SCr, μmol/L** | 72.0 (60.3,81.1) | 65.0 (55.0,77.0) | 60.3 (53.2,71.9) | 58.5 (52.7,66.7) | <0.001 |
| **FPG, mmol/L** | 5.4 (5.2,5.8) | 5.4 (5.1,5.7) | 5.3 (5.1,5.6) | 5.3 (5.0,5.6) | <0.001 |
| **TC, mmol/L** | 4.6 (0.8) | 4.7 (0.8) | 4.8 (0.8) | 5.1 (0.8) | <0.001 |
| **TG, mmol/L** | 1.5 (1.1,2.0) | 1.2 (0.9,1.6) | 1.1 (0.8,1.5) | 1.0 (0.8,1.3) | <0.001 |
| **HDL-C, mmol/L** | 1.1 (0.1) | 1.3 (0.1) | 1.5 (0.1) | 1.8 (0.2) | <0.001 |
| **LDL-C, mmol/L** | 2.64 (0.63) | 2.63 (0.67) | 2.57 (0.67) | 2.52 (0.67) | <0.001 |
| **Non-HDL-C, mmol/L** | 3.51 (0.77) | 3.43 (0.81) | 3.34 (0.81) | 3.29 (0.78) | <0.001 |
| **NHHR** | 3.3 (2.7,3.9) | 2.6 (2.2,3.1) | 2.2 (1.8,2.6) | 1.7 (1.5,2.1) | <0.001 |
| **Diabetes, %** | 471 (5.1%) | 344 (3.7%) | 291 (3.1%) | 218 (2.5%) | <0.001 |
| **Hypertension, %** | 1959 (21.4%) | 1726 (18.4%) | 1629 (17.2%) | 1490 (16.8%) | <0.001 |
| **Hyperuricemia, %** | 2491 (27.2%) | 1840 (19.7%) | 1331 (14.0%) | 885 (10.0%) | <0.001 |
| **MASLD, %** | 3119 (34.0%) | 2139 (22.9%) | 1468 (15.5%) | 761 (8.57%) | <0.001 |

Continuous variables are expressed as mean (standard deviation, SD), median (interquartile range) or n (%). BMI, body mass index; WC, waist circumference; SBP, systolic blood pressure; DBP, diastolic blood pressure; FPG, fasting plasma glucose; TyG, index triglyceride-glucose index; TBil, total bilirubin; DBil, direct Bilirubin; ALB, albumin; PLT, platelet; Hb, hemoglobin; WBC, white blood cell; BUN, blood urea nitrogen; TC, total cholesterol; TG, triglyceride; HDL-C, high-density lipoprotein cholesterol; LDL-C, low-density lipoprotein cholesterol; Non-HDL-C, non-high-density lipoprotein cholesterol; ALT, Alanine aminotransferase; AST, aspartate transaminase; GGT, gamma-glutamyl transpeptidase; UA, uric acid; Cr, creatinine; NHHR, non-high-density lipoprotein cholesterol to high-density lipoprotein cholesterol ratio; MASLD, metabolic dysfunction-associated steatotic liver disease.

**Table S6** Association between NHHR and MASLD in subgroups

| **Subgroups** | **Q1** | **Q2** | **Q3** | **Q4** | ***P* for interaction** |
| --- | --- | --- | --- | --- | --- |
| **Sex** |  |  |  |  | <0.001 |
| Female | 1.00 | 1.58(1.4,1.79) | 2.05(1.79,2.34) | 2.47(2.11,2.9) |  |
| Male | 1.00 | 1.45(1.25,1.68) | 1.63(1.41,1.88) | 1.92(1.65,2.24) |  |
| **Age, years** |  |  |  |  | <0.001 |
| <40 | 1.00 | 1.65(1.44,1.88) | 2.21(1.93,2.54) | 2.27(2.33,3.22) |  |
| 40-60 | 1.00 | 1.51 (1.29,1.76) | 1.67(1.43,1.94) | 1.93(1.63,2.28) |  |
| >60 | 1.00 | 1.41 (1.07,1.85) | 1.60(1.22,2.11) | 1.83(1.36,2.48) |  |
| **BMI** |  |  |  |  | <0.001 |
| <24 | 1.00 | 1.71(1.52,1.92) | 2.17(1.91,2.45) | 2.48(2.15,2.85) |  |
| 24-28 | 1.00 | 1.15 (0.98,1.34) | 1.28(1.10,1.50) | 1.60(1.35,1.89) |  |
| >28 | 1.00 | 2.41 (0.94,6.16) | 2.29(0.9,5.84) | 1.97(0.7,5.54) |  |
| **Hypertension** |  |  |  |  | <0.001 |
| No | 1.00 | 1.66 (1.49,1.85) | 2.10 (1.87,2.34) | 2.48(2.19,2.81) |  |
| Yes | 1.00 | 1.33 (1.1,1.62) | 1.42(1.17,1.73) | 1.68(1.36,2.08) |  |
| **Diabetes** |  |  |  |  | <0.157 |
| No | 1.00 | 1.61(1.46,1.77) | 1.97(1.79,2.18) | 2.33(2.09,2.61) |  |
| Yes | 1.00 | 1.16(0.76,1.77) | 1.13(0.75,1.72) | 1.28(0.81,2.02) |  |
| **Hyperuricemia** |  |  |  |  | 0.005 |
| No | 1.00 | 1.60(1.44,1.77) | 1.95(1.75,2.18) | 2.34(2.06,2.64) |  |
| Yes | 1.00 | 1.40(1.12,1.76) | 1.64(1.32,2.03) | 1.89(1.50,2.38) |  |

Sex, Age, BMI, UA, LDL-C, TG and AST/ALT were adjusted, the model no longer adjust for stratification variables. The results are presented as HRs and 95% CIs. HR, hazard ratio; CI, confidence interval; BMI, body mass index; TG, triglyceride; ALT, alanine aminotransferase; AST, aspartate aminotransferase; UA, uric acid; NHHR, non-high-density lipoprotein cholesterol to high-density lipoprotein cholesterol ratio; MASLD, metabolic dysfunction-associated steatotic liver disease.

**Table S7** Areas under the receiver operating characteristic curves for each lipid parameter in identifying MASLD

|  | **AUC** | **95%CI** | **Best threshold** | **Specificity** | **Sensitivity** | **Youden Index** |
| --- | --- | --- | --- | --- | --- | --- |
| **NHHR** | 0.702 | 0.698-0.707 | 2.536 | 0.597 | 0.714 | 0.311 |
| **HDL-C** | 0.672 | 0.666-0.679 | 1.410 | 0.732 | 0.525 | 0.256 |
| **TyG** | 0.658 | 0.651-0.665 | 7.014 | 0.604 | 0.626 | 0.230 |
| **TG** | 0.649 | 0.642-0.656 | 1.300 | 0.627 | 0.589 | 0.216 |
| **Non-HDL-C** | 0.611 | 0.604-0.618 | 3.210 | 0.590 | 0.574 | 0.164 |

AUC: area under the curve; HDL-C, high-density lipoprotein cholesterol; Non-HDL-C, non-high-density lipoprotein cholesterol; NHHR, non-high-density lipoprotein cholesterol to high-density lipoprotein cholesterol ratio; TyG, index triglyceride-glucose index; TG, triglyceride; MASLD, metabolic dysfunction-associated steatotic liver disease.


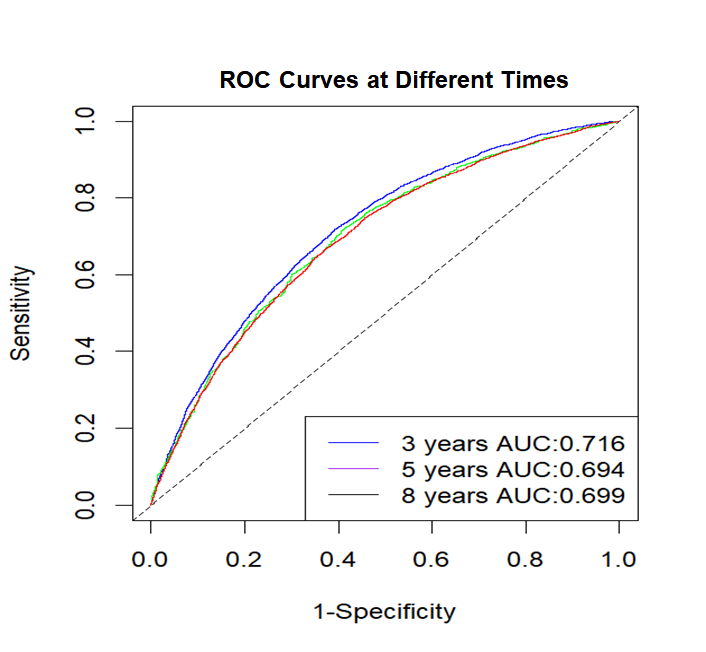


**Figure S1** Time-dependent ROC curves for the prediction of MASLD

ROC, receiver operating characteristic curve; MASLD, metabolic dysfunction-associated steatotic liver disease; AUC, area under the curve.
